# A Study of the Chemical Composition, Acute and Subacute Toxicity of Bulgarian *Tanacetum parthenium* Essential Oil

**DOI:** 10.3390/molecules28134906

**Published:** 2023-06-22

**Authors:** Borislava Lechkova, Diana Karcheva-Bahchevanska, Kalin Ivanov, Velislava Todorova, Niko Benbassat, Nadya Penkova, Pepa Atanassova, Lyudmil Peychev, Petar Hrischev, Zhivko Peychev, Dimitar Terziev, Stanislava Ivanova

**Affiliations:** 1 Department of Pharmacognosy and Pharmaceutical Chemistry, Faculty of Pharmacy, Medical University-Plovdiv, 4002 Plovdiv, Bulgaria; borislava.lechkova@mu-plovdiv.bg (B.L.); diana.karcheva@mu-plovdiv.bg (D.K.-B.); kalin.ivanov@mu-plovdiv.bg (K.I.); velislava.todorova@mu-plovdiv.bg (V.T.); niko.benbasat@mu-plovdiv.bg (N.B.); 2 Department of Anatomy, Histology and Embryology, Faculty of Medicine, Medical University-Plovdiv, 4002 Plovdiv, Bulgaria; nadya.penkova@mu-plovdiv.bg (N.P.); pepa.atanasova@mu-plovdiv.bg (P.A.); 3 Department of Pharmacology, Toxicology and Pharmacotherapy, Faculty of Pharmacy, Medical University-Plovdiv, 4002 Plovdiv, Bulgaria; lyudmil.peychev@mu-plovdiv.bg; 4 Department of Physiology, Faculty of Medicine, Medical University-Plovdiv, 4002 Plovdiv, Bulgaria; petar.hrischev@mu-plovdiv.bg; 5 Department of Medical Informatics, Biostatistics and E-Learning, Faculty of Public Health, Medical University-Plovdiv, 4002 Plovdiv, Bulgaria; zhivko.peychev@mu-plovdiv.bg; 6 Second Department of Internal Diseases, Section of Gastroenterology, Faculty of Medicine, Medical University-Plovdiv, 4002 Plovdiv, Bulgaria; dimitar.terziev@mu-plovdiv.bg

**Keywords:** *Tanacetum parthenium*, essential oil, feverfew, acute and subacute toxicity, hematological, serum biochemical, histological parameters

## Abstract

Background: *Tanacetum parthenium* (L.) Sch.Bip. (*T. parthenium*) is an aromatic perennial plant belonging to the Asteraceae family, also known as feverfew. It is widely distributed in various regions of Europe and other parts of the world. The plant has a rich background in the traditional medicine of many nations and has been used as a remedy for fever, pain, inflammation, asthma, rheumatism, menstrual disorders, etc. Methods: GC–MS analysis was conducted to determine the chemical composition of the isolated essential oil (EO). Using the method proposed by Litchfield and Wilcoxon, the average lethal dose (LD_50_) of the EO on Wistar rats was determined for two routes of administration: oral (p.o.) and intraperitoneal (i.p.). The subacute toxicity of the EO was also tested by oral administration of a daily dose of 1.0 g/kg body weight (BW) for 28 days. The toxicity of the EO was evaluated by observing and evaluating changes in behavior, body weight, basic hematological and serum biochemical parameters, and histopathological changes of the internal organs. Results: Thirty-seven volatile organic compounds representing 94.58% of the total oil composition were tentatively detected in the obtained *T. parthenium* EO. The dominant compounds were camphor (45.47%), *trans*-chrisantenyl acetate (21.65%), camphene (9.48%), and *cis*-isogeraniol (5.42%). The results showed that the EO was not toxic when administered in acute oral doses. The acute mean lethal dose for intraperitoneal administration was LD_50_ i.p. = 2.13 g/kg BW. In the subacute study involving administration of an oral dose of EO for 28 days, there were a number of changes in the hematological and serum biochemical parameters of the blood compared with the control group of animals. However, no symptoms of toxicity, changes in the body weight of the rats, death, or pathological changes in the histological indicators of the examined organs—brain, heart, stomach, liver, spleen and kidney—were found. Extrapolating the results obtained from the rat experiments, we can state that the EO is safe for use in doses below 1 g/kgBW for a period not exceeding one month.

## 1. Introduction

*Tanacetum parthenium* (L.) Sch.Bip. (*T. parthenium*) is an aromatic perennial plant that belongs to the Asteraceae family and is also known as feverfew [1,2]. It is native to the Balkan Peninsula, but also widely distributed in other regions of Europe, North Africa, South and North America, Australia, China, and Japan [1,2]. *Tanaceti parthenii herba* is included in the European Pharmacopoeia [3]. According to the European Pharmacopoeia, the stem is branched, quadrangular, and slightly pubescent with a diameter of up to 5 mm. The leaves are 2–5 cm long, ovate, and yellowish-green. The flowering heads are clustered into broad corymbs consisting of 5–30 flower-heads and their diameter is 12–22 mm. The central flowers are yellow and the peripheral flowers are white [3].

*Tanacetum parthenium* plays an important role in the traditional medicine of different nations and tribes. It has been widely used in folk medicine as a herbal remedy for fever, pain, inflammation, asthma, rheumatism, menstrual disorders, insect bites, infertility, toothache, etc. [4,5,6]. In the southern region of Ecuador, the healers of the Saraguro community have even used the plant to treat supernatural diseases and for psychoactive purposes. In this region, the plant is known as Santa María de huerta [7]. 

In recent years, the interest in this species has increased due to its beneficial effects regarding migraine prophylaxis, although some reports are contradictory [8,9,10,11]. Recently, *T. parthenium* has also been associated with anti-inflammatory and neuromodulatory activity [12].

*Tanacetum parthenium* is a source of sesquiterpene lactones, flavonoids, and essential oils which determine its significant pharmacological activities [2]. Sesquiterpene lactones have been reported to exhibit anticancer [13,14,15,16], anti-inflammatory [13,14], and antioxidant properties [17]. The content of flavonoids and phenolic compounds contributes to the anti-inflammatory [2,18,19] and antioxidant effects [17,20,21]. Essential oil (EO) obtained from *T. parthenium* from Tajikistan has been reported to exhibit a low antioxidant and anti-inflammatory activity and medium cytotoxicity against cancer cell lines in comparison with doxorubicin [5]. Polatoglu et al. (2010) reported on the low-to-medium DPPH scavenging activity of the tested feverfew EO [22]. A study conducted on Iranian *T. parthenium* EO indicated considerable antioxidant capacity [23]. Furthermore, investigations on the activity of the EO against some bacteria and fungi have indicated relatively good antimicrobial properties [22,24,25,26,27]. 

The chemical composition of the EO varies depending on the geographical location, development stage, and morphological parts of the plant. According to different studies and based on GC-MS analysis, the major compounds are camphor (10.3–94%), camphene (1.7–13.74%), and chrysantenyl acetate (4.3–33.8%) [5,22,23,25,26,27,28,29]. Other common constituents in higher amounts are p-cymene, germacrene, and bornylacetate [1,24,30,31,32].

The aim of this study was to analyze the chemical composition of the EO of Bulgarian *T. parthenium* and to investigate in vivo its acute and subacute toxicity in Wistar rats. The current investigation reveals future perspectives for the utilization of the Bulgarian feverfew EO in herbal preparations.

## 2. Results and Discussion

### 2.1. GC-MS Analysis of T. parthenium EO

**Figure 1:**
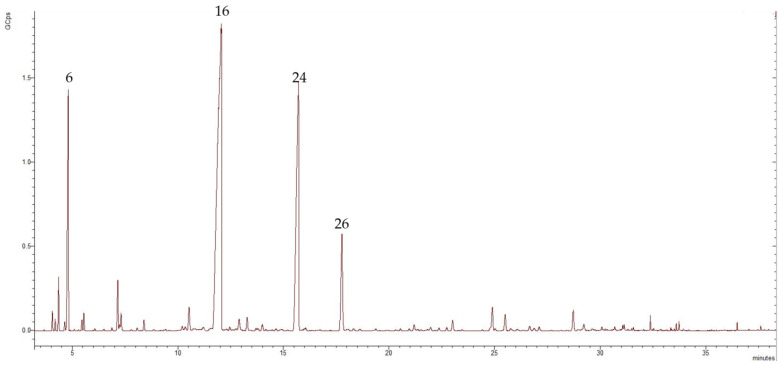
Chromatogram of the isolated *T. parthenium* EO, in which the numbers refer to the following: 6—camphene, 16—camphor, 24—*trans*-chrisantenyl acetate, and 26—*cis*-isogeraniol; GCps refers to giga counts per second.

The obtained EO was analyzed by GC-MS. The chemical composition of the isolated Bulgarian *T. parthenium* EO was tentatively characterized by 37 components, representing 94.58% of the total oil composition. The major volatile compounds were camphor, *trans*-chrisantenyl acetate, camphene, and *cis*-isogeraniol representing 45.47%, 21.65%, 9.48%, and 5.42% of the total oil composition, respectively. Figure 1 shows the chromatogram of the examined EO.

The EO obtained from *T. parthenium* was rich in oxygenated monoterpenes (75.92%), followed by monoterpene hydrocarbons (15.54%), sesquiterpene hydrocarbons (2.76%), oxygenated sesquiterpenes (0.26%), and phenyl propanoid, which had only one representative: eugenol (0.1%). The major volatile organic constituent was the oxygenated monoterpene camphor, which represented 45.47% of the total composition of the oil. Within the oxygenated monoterpene class were *trans*-chrisantenyl acetate (21.65%) and *cis*-isogeraniol (5.42%). Of the monoterpene hydrocarbons, the dominant compound was camphene (9.48%), followed by p-cymene (1.73%) and α-pinene (1.37%). Sesquiterpene hydrocarbons accounted for 2.76% of the total composition of the oil and the main representative was β-farnesene (1.76%). Only a small amount of oxygenated sesquiterpenes were present: 0.26% of the total oil composition. Table 1 presents data regarding the chemical composition of the *T. parthenium* EO showing formulas, retention indices, class terpenes, and percentages of total composition.

To our knowledge, limited data exist regarding the chemical composition of Bulgarian *T. parthenium* EO. The results obtained can be compared with those of other researchers from different geographical regions. A previous study concerning the isolation of *T. parthenium* EO from two different locations in Turkey reported that in the sample collected from the Davutpasa–Istanbul region, the main volatile organic compounds were camphor, *trans*-chrysantenyl acetate, and camphene, representing 49%, 22.1%, and 9.4%, respectively. In contrast, the main compounds detected in the *T. parthenium* EO collected from the Savsat–Ardahan area were camphor (60.8%) and camphene (6.8%) [22]. The main volatile compounds detected in EO from *T. parthenium* collected in Turkey were camphor (56.9%), camphene (12.7%), and p-cymene (5.2%) [1]. The study reported on the chemical composition of *T. parthenium* EO at different developmental stages and found that the main components, camphor, bornyl acetate, and camphene, were present in greater concentrations in the EO obtained from the aerial parts of *T. parthenium* during the flowering stage [27]. It was also reported that in EO obtained from *T. parthenium* samples originating from England and the Netherlands, camphor and chrisantenyl acetate were the main compounds detected [33]. In a study concerning the chemical composition of EO obtained from *T. parthenium* grown in Kosovo, the main compounds found were camphor and camphene [34]. Camphor and camphene were also the main compounds detected in EO from *T. parthenium* collected from Tajikistan [5]. Analyses of EOs from four cultivated *T. parthenium* populations revealed that the predominant volatile compounds were camphor, *trans*-chrysantenyl acetate, and camphene, representing 46.4–47.2%, 22.4–27.3%, and 10.9–12.7%, respectively. The seed material originated from the Netherlands, USA, Italy, and Canada, and the populations were cultivated in Pancevo, Serbia, under identical conditions [28]. Mirjalili et al. (2007) conducted a comparative study on the composition of EO from wild and cultivated feverfew populations in Iran. The oil from the wild population was determined to be a camphor and germacrene-D chemotype, with camphor (50.5%) and germacrene-D (9.2%) as the major components, followed by camphene (7.7%) and (*E*)-sesquilavandulol (4.8%). Chrysantenyl acetate was not detected in this sample [31]. The EO from the cultivated population was characterized by a high amount of camphor (57.6%), chrysantenyl acetate (25.1%), and camphene (4.6%), which correlated with the camphor and chrysantenyl-acetate chemotype [31]. Camphor, chrysantenyl acetate, and camphene predominated in the EOs obtained from *T. parthenium* in Hamedan and Tehran (Iran) [25]. The camphor and chrysantenyl-acetate chemotype of feverfew was also observed in northern Iran. The fraction of farnesol in the analyzed sample was significant at 7.54%, whereas no camphene was detected [23]. In another study from Iran, the major volatile compounds were camphor, *trans*-farnesene, camphene, β-caryophyllene, and chrysanthenone [24]. Investigation of the composition of EO from *T. parthenium* from Egypt revealed camphor and chrysantenyl acetate as the predominant components, with camphene also detected [29]. The main volatile organic compounds detected in *T. parthenium* EO from different geographical regions were camphor, camphene, and chrisantenyl acetate, but the amounts were quite different. The differences in the chemical composition of *T. parthenium* EOs obtained from different geographical regions are due to exogenous and endogenous factors affecting the volatile organic composition of the EOs [35]. Climate, growing stage, and soil type may affect the amounts of terpene in the essential oil [35].

The dominant volatile compound in the analyzed *T. parthenium* EO was camphor. This is a substance present in *Cinnamomum camphora* and other species of the Lauraceae family. Camphor can be obtained from the wood, twigs, and bark of the camphor laurel tree via steam distillation, purification, and sublimation [36,37], and this compound is found in many other EOs (sage, rosemary, basil, etc.) [36]. It is a bicyclic monoterpene that is used in traditional medicine in the form of camphor balm, oil, or cream applied locally to relieve pain associated with inflammation-related disease [36,38]. Camphor is particularly beneficial in the treatment of nasal congestion. Today, it is found in many pharmaceutical products and is also widely used in cosmetics [39]. Examples of the reported biological activities of camphor are: analgesic, antimicrobial, antiviral, anticancer, and antitussive [39,40]. Camphor-based imine derivatives are associated with antiviral activity [38]. In recent years, EOs with a high camphor content have been suggested as potential agents against SARS-CoV-2; however, the results are inconclusive [41,42,43].

Although camphor itself is associated with weak antibacterial, antifungal, and antiviral activity, its toxicity is well documented: 3.5 g of camphor is considered a lethal dose and 2.0 g is associated with toxic effects in adults affecting the gastrointestinal tract, kidneys, and brain [39]. However, cases of camphor poisoning are more often due to accidental ingestion or irrational use rather than its intrinsic toxicity, and it is still considered suitable as a salve for local application when the recommended prescriptions and contraindications are followed [37,43].

Camphene is a secondary metabolite that belongs to the group of monoterpene hydrocarbons [44]. It is a predominant volatile component in essential oils obtained from *Thymus*, *Origanum,* and *Salvia genera* [44]. It has been reported that camphene possesses hepatoprotective, antiviral, and antiacetylcholinesterase inhibitory activities [44]. Camphene has also exhibited significant antioxidant and cytoprotective activity against t-BHP-induced oxidative stress, which suggests its potential use in prophylaxis and therapy for lung inflammation and other oxidative-stress-mediated diseases [45]. Camphene is a component of the EOs obtained from cinnamon, nutmeg, turmeric, cardamom, ginger, etc. It is used as a flavoring and fragrance agent [45]. It has been reported to exhibit expectorant [46], antifungal [47], and antioxidant [48] effects.

Chrysantenyl acetate is believed to play a role in prostaglandin biosynthesis inhibition and therefore may exhibit analgetic effects [28,49]. Together with camphor and camphene, chrysantenyl acetate contributes to the antimicrobial properties of the EO [26]. Chrysantenyl acetate was detected as the main component (49.15%) of the EO from *Artemisia absinthium,* which showed a promising antioxidant activity [50].

### 2.2. Toxicology Results

#### 2.2.1. Evaluation of Acute Toxicity (LD_50_)

The acute toxicity results for *T. parthenium* EO are presented in Table 2 for oral administration and Table 3 for the intraperitoneal route of administration.

No toxic symptoms or signs related to mortality were observed after all doses of the tested plant extract were administered orally. Behavioral reactions were observed for a period of 24 h, and no important changes in behavior, breathing, skin effects, water consumption, impairment in food intake, or temperature were evident.

Although some authors have reported [51,52,53] that large doses of essential oil induce convulsions, there has been no systematic evaluation of the toxic effects of *T. parthenium*. Our investigation showed that the essential oil of the Bulgarian population of *Tanacetum parthenium* is nontoxic when administered to rats as a single oral dose and moderately toxic when administered as a single dose via the intraperitoneal route. The calculated LD_50_ i.p. was 2.13 g/kg BW (1.30 ÷ 3.34); χ^2^ = 4.32; *p* < 0.05; and S_function_ = 2.212.

#### 2.2.2. Subacute Toxicity—Hematological and Serum Biochemical Results 

All animals in the control group treated with Oleum Helianthi and the test group treated with Oleum Helianthi and *T. parthenium* EO survived to the end of the experiment. No signs of clinical toxicity were found in the test group compared with the control group, and there was no statistically significant difference in body weights between the groups.

Table 4 and Table 5 present data from the comparative analysis of hematological and biochemical indicators of the test group, treated with *T. parthenium* EO for 28 days, and those of the control group. With regard to hematological parameters, a significant increase in RBC (control vs. test: 7.74 ± 0.47 × 10^12^/L vs. 8.22 ± 0.42 × 10^12^/L, *p* < 0.05) was observed in the rats treated with *T. parthenium* (Table 4). There were no statistically significant differences between the two groups in terms of the other red blood cell parameters: HGB, HCT, and MCV, (*p* > 0.05). Furthermore, the total WBC count did not differ statistically between the two groups (*p* < 0.05), but there was a difference in the differential blood count. Rats treated with *T. parthenium* showed an increased percentage of neutrophils (control vs. test: 17.07 ± 2.85% vs. 30.59 ± 6.72%, *p* < 0.001) and eosinophils (control vs. test: 0.39 ± 0.17% vs. 0.67 ± 0.30%, *p* < 0.05) and a decrease in lymphocytes (control vs. test: 82.55 ± 2.89% vs. 67.39 ± 6.24%, *p* < 0.001). No statistically significant differences were found regarding basophils and monocytes (*p* > 0.05). Blood cells are formed in the bone marrow via the process of hematopoiesis which is highly regulated. The formation of RBC is controlled by erythropoietin, a hormone produced by the kidneys. Bearing this in mind, and noting the high RBC count in the *T. parthenium*-treated rats, one could discuss the possibility that the EO directly affects erythropoiesis or the secretion of erythropoietin from the kidneys. In our study, we investigated the traditional markers used to evaluate an infectious process such as WBC count, neutrophils, and CRP level. There was no statistically significant difference in WBC and CRP between the two study groups (*p* > 0.05). Compared with the control group, circulating neutrophils and eosinophils increased and lymphocytes dropped in the test group of animals treated with *T. parthenium* EO. It is known that neutrophilia may occur with or without an elevated WBC count. Factors such as physical activity and stress increase the risk of increasing neutrophils and decreasing lymphocytes. For many years, researchers have sought an easily measurable and accessible parameter to determine the intensity of stress and/or systematic inflammation in critically ill individuals [54]. According to data from clinical studies, the inflammatory/immune response to stress can be effectively characterized by the percentage neutrophil-to-lymphocyte ratio [54,55]. In addition, it should be noted that it is appropriate to monitor the WBC count and differential count of leukocytes dynamically, because it is difficult to reach a final conclusion about the reasons that led to the observed changes based on a single examination of these indicators. 

In our study, to assess the renal function of the experimental rats, we examined the serum concentration of the nonprotein nitrogen-containing substances creatinine, urea, and uric acid (Table 5). The homeostatic function of the kidneys was assessed by measuring sodium, potassium, total calcium, inorganic phosphorus, and total magnesium. There were no differences between the control and test groups in mean values of urea (control vs. test: 2.99 ± 0.56 mmol/L vs. 2.45 ± 0.82 mmol/L, *p* > 0.05), uric acid (control vs. test: 135.50 ± 17.43 μmol/L vs. 120.50 ± 30.27 μmol/L, *p* > 0.05), magnesium (control vs. test: 1.05 ± 0.03 mmol/L vs. 0.96 ± 0.33 mmol/L, *p* > 0.05), inorganic phosphate (control vs. test: 1.78 ± 0.12 mmol/L vs. 1.70 ± 0.12 mmol/L, *p* > 0.05), sodium (control vs. test: 140.70 ± 0.82 mmol/L vs. 141.40 ± 1.51 mmol/L, *p* > 0.05), or potassium (control vs. test: 5.72 ± 0.60 mmol/L vs. 5.96 ± 0.46 mmol/L, *p* > 0.05). Compared with the control group, the *T. parthenium* EO-treated animals had statistically significant higher levels of serum creatinine (control vs. test: 30.60 ± 2.99 μmol/L vs. 34.30 ± 3.50 μmol/L, *p* < 0.05) and total calcium (control vs. test: 2.24 ± 0.04 mmol/L vs. 2.39 ± 0.06 mmol/L, *p* < 0.001). High serum creatinine in *T. parthenium* EO-treated rats suggests possible reversible damage to the nephron and more specifically to its filtration function. The balance of calcium in the body depends on its absorption in the intestines, exchange between blood and bones, and its excretion from the body [56]. The bone and kidney are the two organs that determine the serum calcium level in the fasting state. Intestinal calcium absorption after a meal induces a transient increase in serum calcium. Furthermore, the concentration of total serum calcium depends on the concentration of albumin. According to our data, the group treated with *T. parthenium* had statistically significant higher levels of albumin than the control group (control vs. test: 30.70 ± 1.16 g/L vs. 33.80 ± 1.81 g/L, *p* < 0.001), and it should be noted that the high calcium level in this instance may not be directly related to the administration of EO. 

The excretory function of the liver was assessed by examining total bilirubin. Compared to the control group, the group treated with *T. parthenium* EO had statistically significant higher levels of serum bilirubin (control vs. test: 3.98 ± 0.96 μmol/L vs. 6.29 ± 1.74 μmol/L, *p* < 0.01). In addition, the two groups differed in their mean values of total cholesterol (control vs. test: 1.70 ± 0.17 mmol/L vs. 2.29 ± 0.22 mmol/L, *p* < 0.001) and triglycerides (control vs. test: 0.51 ± 0.12 mmol/L vs. 0.79 ± 0.13 mmol/L, *p* < 0.001). Higher levels of serum bilirubin, cholesterol, and triglycerides in *T. parthenium*-treated rats may suggest an impaired liver excretory function. Based on our gas-chromatographic studies, the likely reason for these elevated values is the high concentration of camphor. Several other studies have suggested liver damage associated with camphor intake, although the exact mechanism of its toxicity is not fully established [57,58,59,60,61,62]. On the other hand, hepatoprotective properties are attributed to certain other components of the EO. Camphene exhibits a protective effect against nimesulide-induced hepatotoxicity [63]. Camphene also prevents hepatic steatosis and insulin resistance in mice [64]. The hepatoprotective activity of *trans*-β-farnesene was observed by Vinholes et al. (2014) [65]. Para-cymene can reduce hepatic injury [66]. However, the two groups in our study did not differ in mean values of ALT, ALP, and platelets, which indicates that there are insufficient data regarding serious liver injury and malfunction. Compared to the control group, the *T. parthenium*-treated animals had a statistically significant lower AST (control vs. test: 245.90 ± 35.36 IU/L vs. 172.90 ± 23.83 IU/L, *p* < 0.001). However, this result has no obvious diagnostic value with regard to possible liver damage because AST is not an enzyme specific to the liver and is only of importance if the values are elevated.

The cardiac MB isoenzyme of CK in serum is a typical biochemical marker of myocardial necrosis. From this point of view, the absence of a statistically significant difference between CK, and in particular CK-MB, between the two groups indicates that there is no evidence of cardiac involvement in the group treated with *T. parthenium*. This statement is supported by the absence of pathological changes in the histological findings from the myocardia of the animals treated with *T. parthenium* EO.

Further research is required to explain the statistically significant lower blood sugar levels found in the group of animals treated with *T. parthenium* EO (control vs. test: 7.85 ± 0.87 mmol/L vs. 6.93 ± 0.74 mmol/L, *p* < 0.05).

#### 2.2.3. Subacute Toxicity—Histological Results

**Figure 2:**
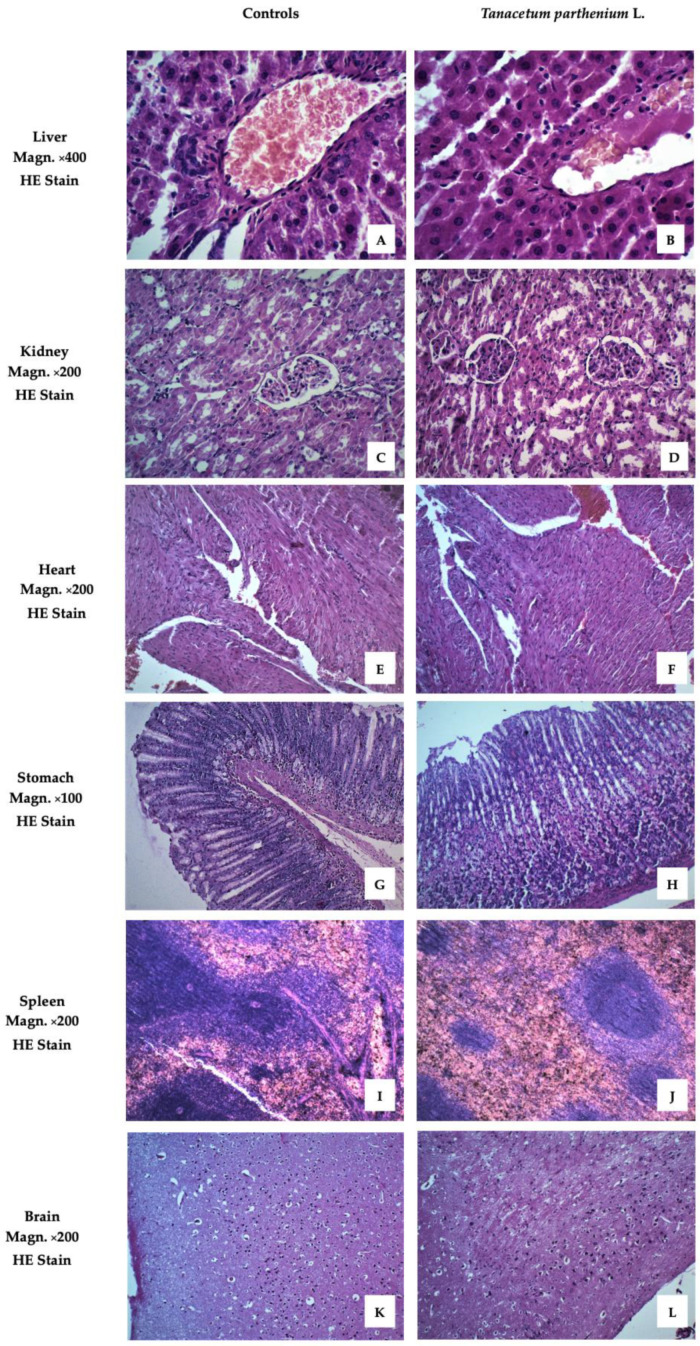
Liver, kidney, heart, stomach, spleen, and brain of animals from the tested group, stained with hematoxylin and eosin (HE). (**A**) Liver with normal structure. The focus on v. centralis with the radiating hepatocytes in its center demonstrates the correct cyto- and angio-architectonics of the organ. Certain hepatocytes have a more intense staining of the cytoplasm. (**B**) Liver without signs of pathological damage. Anastomosing tufts of hepatocytes around v. centralis. Hepatocytes with well-stained cytoplasm, some of them binucleate. Scarce connective tissue around interlobular artery, vein, and bile duct. (**C**) Kidney with normal structure. Renal cortex with dense sections of the convoluted parts of proximal and distal tubules. Malpighian corpuscles, which have a clearly visible single-layer squamous epithelium on the outer layer of Baumann’s capsule. (**D**) Kidney with normal structure. Malpighian bodies in the cortical part with narrow slit-like spaces between the two sheets of Baumann’s capsule. Sections of curved parts of proximal tubules predominate. They have a smaller, irregular lumen and cells with well-stained cytoplasm, a sign of intense functional activity. Distal tubules with lighter cytoplasm and cubic cells. (**E**) Myocardium with normal structure. Cardiomyocytes organized in layers in different directions. A longitudinal section shows the characteristic shape of the cells forming a functional syncytium. Certain cardiomyocytes have more pronounced eosinophilia. (**F**) Myocardium with normal structure. Dense layers of cardiomyocytes in different directions. Larger caliber blood vessels with a well-structured endothelium are also visible between some of them. (**G**) Stomach with normal structure. Fundus mucosa with preserved covering epithelium and closely spaced major glands. The principal pepsinogen-producing basophilic cells occupy the bottom of the glands. Rounded, eosinophilic cells producing hydrochloric acid are well represented in the central areas of the mucosa. (**H**) Stomach with normal structure. Corpus mucosa with dense coverings of major glands, well bounded by lamina muscularis mucosae. An area with a large number of parietal cells occupying the central region of the glands. (**I**) Spleen with normal structure. Well-differentiated white and red pulp. Formed trabeculae and active splenic lymphatic follicles (Malpighian corpuscles) with well-defined zones pierced eccentrically by a lymphonoduli. (**J**) Spleen with normal structure. Red pulp that predominates on the particular slice over white pulp. Certain splenic follicles (Malpighian corpuscles) of smaller size are without distinguishable zones. (**K**) Cerebral cortex with normal structure. Preserved layered construction. Clearly visible superficial molecular layer with predominant nerve processes and a small number of small neurons scattered among it. Underlying outer granular and pyramidal layers flowing into each other. (**L**) Cerebral cortex with normal structure. Well-formed molecular layer and outer granular layer of a large number of closely spaced neurons, both granular and pyramidal.

Figure 2 presents images of the examined organs from Wistar rats treated with *T. parthenium* EO. These demonstrated no pathological anomalies.

The images showed no differences between the livers of animals from the treated and control groups. The liver lobules were clearly defined, with normal structures of central veins and triads. The hepatocytes exhibited no alterations from normal morphology.

The cortex and medulla of the kidneys retained their normal morphology. No changes were observed in the microstructure of the glomerulus or any of the canal systems.

To evaluate of the effect of drug toxicity on the heart, histological and cytological studies of the heart are required. No pathological changes in the hearts of the two groups were found. The heart is mainly composed of cardiomyocytes and a small amount of connective tissue. Cardiomyocytes join together to form the myocardium.

The stomachs of animals from the group treated with *T. parthenium* EO showed no differences compared to the control group. The innermost layer of the wall of the organ, the gastric mucosa, was well preserved. This is formed by a layer of surface epithelium (a simple columnar epithelium) and an underlying lamina propria and muscularis mucosae. The surface epithelium lines the inside of the stomach as surface mucous cells. Gastric glands are found throughout the inner surface of the stomach and open into the base of the gastric pits.

The parenchyma of the spleen revealed a normal microscopic structure with white and red pulp. The white pulp, the main lymphoid tissue of the spleen, was demonstrated as a minor accumulation of lymphocytes around arterial vessels.

The brain sample images of the experimental animals showed a normal structure and no remodeling of the cerebral cortex.

An examination of the outer morphology and histological structure of the main organs responsible for the elimination of xenobiotics and detoxication of the organism revealed no toxic effect or dysfunctionality associated with the investigated essential oil plant.

In the last two decades, research on herbs with potential uses in medicine has thrived [67,68,69,70,71,72,73]. The results demonstrated in the current study represent an important key point for future research regarding the short-term use of *T. parthenium* EO. We can claim that this EO is safe for use in doses less than 1 g/kg BW for a period no longer than 1 month.

## 3. Materials and Methods

### 3.1. Plant Materials

Aerial parts of the *T. parthenium* L. were collected in South Bulgaria. The plant was authenticated by the authors in accordance with the European Pharmacopoeia [3].

### 3.2. Chemicals and Reagents

Hexane of analytical grade was purchased from Thermo Fisher Scientific GmbH (Bremen, Germany) and was used for the dilution of the EO. The following hydrocarbons were used to determine the retention indices (RIs): nonane (99%), decane (≥99%), undecane (≥99%), dodecane (99%), tridecane (≥99%), tetradecane (≥99%), hexadecane (≥99%), heptadecane (99%), octadecane (99%), nonadecane (99%), eicosane (99%), and heneicosane (≥99.5%). These were purchased from Merck KGaA (Darmstadt, Germany).

### 3.3. Isolation of the Essential Oil 

The air-dried flowering aerial parts (100 g) of the plant were subjected to hydrodistillation for 4 h using a Clevenger-type apparatus to obtain the essential oil. The collected oil was dried over anhydrous sodium sulfate and stored in dark glass vials at 4 °C until GC-MS analysis.

### 3.4. Chromatographic Condition

The obtained EO was analyzed using gas chromatography with mass spectrometry (GC–MS). The GC–MS analyses were carried out using a Bruker Scion 436-GC SQ MS (Bremen, Germany). The ionization energy for the mass was 70 eV, and the mass spectra were collected in the range of *m*/*z* 50–250 in full scan mode. The column analysis used was a Bruker BR-5ms fused silica capillary (0.25-μm film thickness and 15 m × 0.25 mm i.d.). The injector was split/splitless with a split ratio of 1:20, and the injection volume was 1 μL. The temperature of the oven was initially held at 45 °C for 1 min, increased to 130 °C at 3 °C/min, increased to 250 °C at 15 °C/min, and then held for 1 min. The carrier gas was helium with a constant flow rate of 1 mL/min. The temperatures of the detector and injector were 300 and 250 °C, respectively. The collected MS spectra were compared with spectral data and RIs in the Wiley NIST11 Mass Spectral Library (NIST11/2011/EPA/NIH) and data from the literature. The determination of retention indices in the GC-MS analysis of the essential oil followed a standardized procedure. A mixture of n-alkane standards (C_9_–C_20_) was injected into the gas chromatograph under the same chromatographic conditions as the EO samples. Subsequently, the retention index of each compound was calculated using the retention times of the n-alkane standards and the retention times of the individual volatile compounds in the essential oil. The resulting RI values were then compared to the RI values of known compounds in a reference database. 

### 3.5. Toxicology

Permission to use animals in the experiment was obtained from the Food Safety Agency at the Bulgarian Ministry of Agriculture and Food (No. 238/2019, valid until 11 September 2024) and was formally approved by the Ethical Committee on Human and Animal Experimentation of the Medical University, Plovdiv. All procedures were conducted in accordance with the European Community Council directives: 86/609/EEC. 

#### 3.5.1. Acute Toxicity Study of *T. parthenium* EO to Determine LD_50_

A total of 60 male Wistar rats (180.0 ÷ 200.0 g BW), equally divided into 10 groups, were administered a single dose of *T. parthenium* by two routes of administration—p.o. and i.p. Groups No. I, II, III, IV, and V were administered EO p.o. by gastric tube in doses of 1.0 g/kg BW, 3.0 g/kg BW, 5.0 g/kg BW, 8.0 g/kg BW, and 10.0 g/kg BW, respectively. Groups VI, VII, VIII, IX, and X were treated i.p. with doses of 1.0 g/kg BW, 1.75 g/kg BW, 2.0 g/kg BW, 3.0 g/kg BW, and 5.0 g/kg BW, respectively. General behavior and mortality were observed for up to 24 h.

The results for LD_50_-values were evaluated according to the Litchfield and Wilcoxon method [74].

#### 3.5.2. Subacute Toxicity Study—Hematological and Serum Biochemical Evaluations

The experimental animals, 20 white male Wistar rats weighing 180–200 g, were divided into two groups of 10 animals. The animals were treated once a day orally by gastric tube for 28 days as follows: the control group received Oleum Helianthi at a dose of 1.0 g/kgBW, and the test group received *T. parthenium* EO dissolved in Oleum Helianthi at a dose of 1.0 g/kgBW. From the start of the experiment, the body weight of each animal was determined weekly.

The animals were housed in the university vivarium and maintained under standard laboratory conditions: a 12:12 h (light/dark) cycle at a temperature of 23 ± 1 °C and access to water and laboratory chow ad libitum. To avoid changes in body weight, a balanced diet was administered to rodents.

At the end of the experiment on the 28th day, two hours after the administration of Oleum Helianthi and *T. parthenium* EO, respectively, for the controls and the tested group, blood samples were drawn from the tails for the examination of hematological and serum biochemical parameters. Hematological indicators were determined using an automatic veterinary analyzer (RT-7600 Vet, GuangZhou, China). Hemoglobin (HGB), red blood cells (RBC), hematocrit count (HCT), mean erythrocyte volume (MCV), white blood cell (WBC) count, platelets (PLT), and the differential count of leukocytes were examined. Biochemical parameters were performed using a Chemray-120 Automated Chemistry Analyzer (GuangZhou, China). Serum concentrations of glucose, aspartate aminotransferase (AST), alanine aminotransferase (ALT), alkaline phosphatase (ALP), lactate dehydrogenase (LDH), creatine kinase (CK), creatine kinase-MB (CK-MB), alpha-amylase, total bilirubin, total cholesterol, HDL cholesterol, LDL cholesterol, triglycerides, total protein, albumin, C-reactive protein (CRP), rheumatoid factor, urea, creatinine, uric acid, iron, total iron binding capacity, sodium, potassium, total calcium, inorganic phosphate, and total magnesium were analyzed.

#### 3.5.3. Subacute Toxicity Study—Histological Evaluation

On the 29th day of the experiment, after an overnight fast (8 h), the animals were euthanized via anesthesia (thiopental; 100 mg/kg BW). The brain, heart, stomach, liver, spleen, and kidney were then removed from the animals and placed in formalin for fixation to preserve the tissue. The tissues were then embedded in paraffin blocks for histological evaluation. The latter were cut using a Leica 2055 Autocut microtome into 5 µm-thick slices and stained with hematoxylin and eosin (H&E). This staining allows a general assessment of organic structure and pathological changes. The obtained samples were observed using an Olympus BX 51 light microscope (Tokyo, Japan) and photomicrographs were delivered through the microscope camera.

#### 3.5.4. Statistical Analysis

Data for subacute toxicity were expressed as mean ± standard deviation (mean ± SD). The data were analyzed using the SPSS v.24 program for Windows by one-way ANOVA followed by Tukey’s multiple comparison test. *p*-values of less than 0.05 were considered to be statistically significant.

## 4. Conclusions

From the EO obtained from Bulgarian *T. parthenium,* we have tentatively identified volatile organic compounds representing 94.58% of the total oil composition. These compounds were from the following classes: monoterpene hydrocarbons, oxygenated monoterpenes, phenylpropanoids, sesquiterpene hydrocarbons, and oxygenated sesquiterpenes. The dominant terpene class was monoterpene hydrocarbons, with camphor as the major compound representing 45.47% of the total composition of the oil. This study revealed significant findings regarding the acute and subacute toxicity profile of *T. parthenium* EO. The results showed that the EO was not toxic when administered in acute oral doses. The acute mean lethal dose for intraperitoneal administration was LD_50_ i.p. = 2.13 g/kg BW. Extrapolating the results obtained from the rat experiments, we can claim that the EO is safe for use in doses of less than 1 g/kg BW for a period no longer than 1 month. Future chronic toxicity experiments using EO in experimental animals will confirm whether its long-term use is safe or could cause cumulative organ toxicity with damage to internal organs, particularly the liver or kidneys.

## Figures and Tables

**Table 1:** The chemical composition data obtained from GC-MS analysis of *T. parthenium* EO.

No	Compound	Formula	RI	Class Terpene	% of Total
1	Santolina triene	C_10_H_16_	918	MH	tr
2	Tricyclene	C_10_H_16_	928	MH	0.48
3	α-Thujene	C_10_H_16_	931	MH	0.29
4	α-Pinene	C_10_H_16_	936	MH	1.37
5	2,4(10)-Thujadiene	C_10_H_14_	943	MH	0.30
6	Camphene	C_10_H_16_	947	MH	9.48
7	Sabinene	C_10_H_16_	964	MH	0.31
8	β-Pinene	C_10_H_16_	966	MH	0.50
9	α-Terpinene	C_10_H_16_	1000	MH	0.10
10	p-Cymene	C_10_H_14_	1007	MH	1.73
11	D-limonene	C_10_H_16_	1012	MH	0.61
12	γ-Terpinene	C_10_H_16_	1038	MH	0.37
13	Linalool	C_10_H_18_O	1088	MO	0.18
14	α-Cyclocitral	C_10_H_16_O	1093	MO	1.07
15	*cis*-p-Menth-2-en-1-ol	C_10_H_18_O	1110	MO	0.16
16	Camphor	C_10_H_16_O	1132	MO	45.47
17	Pinocarvone	C_10_H_14_O	1142	MO	0.13
18	endo-Borneol	C_10_H_18_O	1153	MO	0.65
19	Terpinen-4-ol	C_10_H_18_O	1163	MO	0.54
20	Myrtenal	C_10_H_14_O	1177	MO	0.11
21	α-Terpineol	C_10_H_18_O	1181	MO	0.32
22	*cis*-Carveol	C_10_H_16_O	1185	MO	tr
23	Piperitol	C_10_H_18_O	1197	MO	0.10
24	*trans*-Chrisantenyl acetate	C_12_H_18_O_2_	1225	MO	21.65
25	Carvone	C_10_H_14_O	1231	MO	tr
26	*cis*-Isogeraniol	C_10_H_18_O	1276	MO	5.42
27	p-Cymen-7-ol	C_10_H_14_O	1290	MO	0.12
28	Carvacrol	C_10_H_14_O	1299	MO	tr
29	Eugenol	C_10_H_12_O_2_	1347	PP	0.1
30	α-Copaene	C_15_H_24_	1368	SH	0.1
31	Caryophyllene	C_15_H_24_	1410	SH	0.51
32	β-Farnesene	C_15_H_24_	1458	SH	1.26
33	Germacrene D	C_15_H_24_	1473	SH	0.76
34	α-Selinene	C_15_H_24_	1478	SH	0.13
35	Globulol	C_15_H_26_O	1576	SO	0.12
36	Humulane-1,6-dien-3-ol	C_15_H_26_O	1616	SO	0.14
37	Longipinocarveol, trans	C_15_H_24_O	1628	SO	tr
	Class terpenes				
	Monoterpenes hydrocarbons—MH				15.54
	Monoterpenes oxygenated—MO				75.92
	Phenylpropanoids—PP				0.1
	Sesquiterpene hydrocarbons—SH				2.76
	Sesquiterpene oxygenated—SO				0.26
	Total identified				94.58

tr—traces (<0.05%); RI—retention index. The data represent the mean of three independent samples. The standard error of the mean does not exceed 2%, and this has been removed to simplify the reporting data.

**Table 2:** Results for oral LD_50_ of *T. parthenium* EO in rats.

Group	Dose (g/kg BW)	D/T	Dead Rats (%)	Symptoms of Toxicity and Death until 24 h
Gr. I	1	0/6	0	None
Gr. II	3	0/6	0	None
Gr. III	5	0/6	0	None
Gr. IV	8	0/6	0	Hypoactivity
Gr. V	10	0/6	0	Hypoactivity. No symptoms of toxicity. No mortality.

None—no symptoms observed up to 24 h. D/T—number of dead rats/number of treated rats.

**Table 3:** Results for intraperitoneal LD_50_ of *T. parthenium* EO in rats.

Group	Dose (g/kg BW)	D/T	Dead Rats (%)	Toxic Effect Up to 24 h
Gr. VI	1.0	0/6	0	10 min after application of EO, limbs were stretched forward. Periodic jumps. Drumming with forelimbs. Onset of mild convulsions after 1 h, which disappeared after a further 2 h. Animals were alive until the 24th hour.
Gr. VII	1.75	3/6	50.0	5 min after the injection of EO, drumming with the front limbs and increasing convulsions with lying down. Death of 3 animals after 1 h.
Gr. VIII	2.0	4/6	66.8	5 min after the injection of EO, light twitching of the limbs, drumming with the front limbs, increasing convulsions with lying down. After 1 h, 4 animals die.
Gr. IX	3.0	5/6	83.3	5 min after the injection of the essential oil, slight twitching of the limbs, drumming with the front limbs, increasing convulsions with lying down, making sounds, and breathing difficulties. Death of 5 animals after 1 h.
Gr. X	5.0	4/6	66.8	Erection of hind limbs and drumming of fore limbs, erection of tail, tonic–clonic seizures, recumbency, and death of 4 rats after 1 h.

D/T—number of dead rats/number of treated rats.

**Table 4:** Hematological parameters in rats treated with *T. parthenium* EO for 28 days.

Parameter	*n*	Control (Mean ± SD)	*T. parthenium* (Mean ± SD)	*p*-Value
RBC (×10^12^/L)	10	7.74 ± 0.47	8.22 ±0.42	*p* < 0.05
MCV (fl)	10	49.20 ± 0.79	48.38 ± 0.74	ns
HGB (g/L)	10	132.20 ± 11.07	139.00 ± 6.24	ns
HCT (%)	10	0.39 ± 0.03	0.41 ± 0.02	ns
WBC (×10^9^/L)	10	4.53 ± 0.6450	4.12 ±1.27	ns
Neutrophils (%)	10	17.07 ± 2.85	30.59 ± 6.72	*p* < 0.001
Lymphocytes (%)	10	82.55 ± 2.89	67.39 ± 6.24	*p* < 0.001
Basophiles (%)	10	0.52 ± 0.16	0.62 ± 0.19	ns
Eosinophils (%)	10	0.39 ± 0.17	0.67 ± 0.30	*p* < 0.05
Monocytes (%)	10	0.86 ± 0.12	0.79 ± 0.13	ns
PLT (×10^9^/L)	10	1110.00 ± 60.75	1171.10 ± 164.89	ns

ns—no statistical difference, *p* > 0.05.

**Table 5:** Biochemical parameters in rats treated with *T. parthenium* EO for 28 days.

Parameter.	*n*	Control (Mean ± SD)	*T. parthenium* (Mean ± SD)	*p*-Value
Glucose (mmol/L)	10	7.85 ± 0.87	6.93 ± 0.74	*p* < 0.05
AST (IU/L)	10	245.90 ± 35.36	172.90 ± 23.83	*p* < 0.001
ALT (IU/L)	10	54.10 ± 7.96	43.90 ± 10.50	ns
ALP (IU/L)	10	415.43 ± 82.17	258.29 ± 45.07	*p* < 0.01
CK (IU/L)	10	2214.60 ± 339.62	2167.10 ± 384.21	ns
CK-MB (IU/L)	10	2970.70 ± 581.42	2436.70 ± 524.37	ns
LDH (IU/lL	10	2635.80 ± 122.86	2415.60 ± 357.79	ns
Total billirubin (μmol/L)	10	3.98 ± 0.96	6.29 ± 1.74	*p* < 0.01
Total cholesterol (mmol/L)	10	1.70 ± 0.17	2.29 ± 0.22	*p* < 0.001
HDL-cholest. (mmol/L)	10	1.01 ± 0.06	1.44 ± 0.07	*p* < 0.001
LDL-cholest. (mmol/L)	10	0.38 ± 0.04	0.35 ± 0.07	ns
Triglycerides (mmol/L)	10	0.51 ± 0.12	0.79 ± 0.13	*p* < 0.001
Total protein (g/L)	10	65.90 ± 2.28	67.30 ± 4.83	ns
Albumin (g/L)	10	30.70 ± 1.16	33.80 ± 1.81	*p* < 0.001
CRP (mg/L)	10	0.76 ± 0.64	0.84 ± 0.36	ns
Creatinin (μmol/L)	10	30.60 ± 2.99	34.30 ± 3.50	*p* < 0.05
Urea (mmol/L)	10	2.99 ± 0.56	2.45 ± 0.82	ns
Uric acid (μmol/L)	10	135.50 ± 17.43	120.50 ± 30.27	ns
Iron (μmol/L)	10	48.68 ± 6.11	51.11 ± 6.40	ns
Sodium (mmol/L)	10	140.70 ± 0.82	141.40 ± 1.51	ns
Potassium (mmol/L)	10	5.72 ± 0.60	5.96 ± 0.46	ns
Total calcium (mmol/L)	10	2.24 ± 0.04	2.39 ± 0.06	*p* < 0.001
Total magnesium (mmol/L)	10	1.05 ± 0.03	0.96 ± 0.33	ns
Inorganic phosphate (mmol/L)	10	1.78 ± 0.12	1.70 ± 0.12	ns

ns—no statistical difference, *p* > 0.05.

## Data Availability

Not applicable.

## Sample Availability

Samples of the essential oil are not available.
